# Comparison of Frailty Screening Instruments in the Emergency Department

**DOI:** 10.3390/ijerph16193626

**Published:** 2019-09-27

**Authors:** Rónán O’Caoimh, Maria Costello, Cliona Small, Lynn Spooner, Antoinette Flannery, Liam O’Reilly, Laura Heffernan, Edel Mannion, Anna Maughan, Alma Joyce, D. William Molloy, John O’Donnell

**Affiliations:** 1 Clinical Sciences Institute, National University of Ireland, Galway, Costello Road, H91 TK33 Galway City, Ireland; m.costello13@gmail.com (M.C.); cliona.small@gmail.com (C.S.); antoinettef1988@gmail.com (A.F.); edel.mannion@hse.ie (E.M.); 2 Department of Geriatric and Stroke Medicine, University Hospital Galway, Newcastle Rd, H91 YR71 Galway City, Ireland; W.Molloy@ucc.ie; 3 Centre for Gerontology and rehabilitation, University College Cork, St Finbarr’s Hospital, Douglas Road, T12 XH60 Cork City, Ireland; lynn.spooner@hse.ie; 4 Mercy University Hospital, Grenville Place, T12 WE28 Cork City, Ireland; 5 Department of Emergency Medicine, University Hospital Galway, Newcastle Rd, H91 YR71 Galway City, Ireland; oreillyliam6@gmail.com (L.O.); lauraheffernan@rcsi.ie (L.H.); johnj.odonnell@hse.ie (J.O.); 6 Primary, Community and Continuing Care, Shantalla Health Centre, Costello Rd, H91 YR71 Galway City, Ireland; anna.maughan@hse.ie (A.M.); alma.joyce@hse.ie (A.J.)

**Keywords:** older people, frailty, emergency department, screening, sensitivity, specificity

## Abstract

Early identification of frailty through targeted screening can facilitate the delivery of comprehensive geriatric assessment (CGA) and may improve outcomes for older inpatients. As several instruments are available, we aimed to investigate which is the most accurate and reliable in the Emergency Department (ED). We compared the ability of three validated, short, frailty screening instruments to identify frailty in a large University Hospital ED. Consecutive patients aged ≥70 attending ED were screened using the Clinical Frailty Scale (CFS), Identification of Seniors at Risk Tool (ISAR), and the Programme on Research for Integrating Services for the Maintenance of Autonomy 7 item questionnaire (PRISMA-7). An independent CGA using a battery of assessments determined each patient’s frailty status. Of the 280 patients screened, complete data were available for 265, with a median age of 79 (interquartile ±9); 54% were female. The median CFS score was 4/9 (±2), ISAR 3/6 (±2), and PRISMA-7 was 3/7 (±3). Based upon the CGA, 58% were frail and the most accurate instrument for separating frail from non-frail was the PRISMA-7 (AUC 0.88; 95% CI:0.83–0.93) followed by the CFS (AUC 0.83; 95% CI:0.77–0.88), and the ISAR (AUC 0.78; 95% CI:0.71–0.84). The PRISMA-7 was statistically significantly more accurate than the ISAR (*p* = 0.008) but not the CFS (*p* = 0.15). Screening for frailty in the ED with a selection of short screening instruments, but particularly the PRISMA-7, is reliable and accurate.

## 1. Introduction

The risk-stratification of older adults presenting to the Emergency Department (ED) is useful to target interventions that reduce their risk of adverse healthcare outcomes [1,2], particularly where frailty is identified [3]. This is challenging because frail patients often present atypically [4,5], and acutely unwell hospitalised older adults may appear frailer than their baseline suggests [6], meaning that resource-intensive, evidence-based interventions such as comprehensive geriatric assessment (CGA) [7] can be difficult to apply. Demographic change is expected to heighten this with a greater number of older and frail patients presenting to EDs [8]. Although older adults represent only between 10%–30% of those presenting to the ED [9,10], those who are frail represent up to 60% of older attendees [11]. Frail older patients have higher ED conversion rates [10] that result in longer admissions [12] with a greater likelihood of readmission [13] and higher inpatient mortality [12]. Further, these patients also represent an increasing proportion of unplanned admissions (unscheduled care) with the ED often seen by patients themselves as the main entry point to acute care [14].

Although the determination of frailty status at admission is important in predicting these outcomes [15], few older adults receive this risk-stratification [16]. The most frequently used frailty screening instruments have short administration times and are considered largely feasible and acceptable to use by ED staff [17,18]. Despite this, a recent systematic review showed very low completion rates of frailty instruments in ED with no study covering more than half (52%) of those potentially eligible for screening [19]. The reasons for this are likely multi-factorial, related to resource pressures and knowledge and training deficits [20] among staff in increasingly busy EDs with an older, frailer, and more complex case-mix [21]. In addition, while a plethora of frailty tools are available [22], risk-stratification in the ED is mainly limited to instruments not designed to measure frailty and by the reliability, diagnostic accuracy, sensitivity and specificity of these instruments [23].

The British Geriatric Society (BGS) [24] recommends opportunistic identification of frailty and suggests the Programme on Research for Integrating Services for the Maintenance of Autonomy 7 item (PRISMA-7) questionnaire [25] among others, as a simple frailty assessment. Although shown to be particularly sensitive in identifying frailty among community-dwellers [26,27], it has not been validated in the ED. As the allocation of limited and time-intensive resources such as CGA is contingent on the robustness of the initial screening and identification [23], it is important to select these based on appropriate evidence.

Given that early identification of frailty in acute care settings allows the rapid allocation of CGA, which is known to reduce mortality and institutionalisation among older people [7], we aimed to compare the clinimetric properties of three commonly used frailty and risk-prediction screening instruments to correctly identify frailty after initial presentation to ED triage. Further, as most studies have not used consecutive sampling [23] due to the challenges set by data collection in the ED environment, data were gathered continuously 24 h per day over a two-week period.

## 2. Materials and Methods

### 2.1. Participants

Consecutive older adults aged ≥70 years were screened at triage after arrival to a large university hospital ED in the West of Ireland. In 2016, there were 64,096 attendances, with 9407 aged ≥70 (14.7%). Screening was performed, where possible 24 h/day Monday to Sunday inclusive, over a two-week period in March 2016. During the period of data collection all patients referred to the acute medical assessment unit were initially triaged in ED. Those deemed medically unstable based on a Manchester Triage System (MTS) score of one were excluded. This scoring system considers five priority levels at triage: immediate (level 1), very urgent (level 2), urgent (level 3), standard (level 4), and non-urgent (level 5) [28]. Similarly, those brought directly to the ED resuscitation room, cardiac care, or intensive care units, irrespective of MTS score, were excluded unless this was for logistical reasons. Residents in nursing homes, where the prevalence of frailty or pre-frailty is over 90% [29] were also excluded. Ethics approval was obtained from the local ethics committee of Galway University Hospitals (reference number *C*.*A.* 1429) and patients provided informed written consent. Where deemed unable, verbal assent was sought.

### 2.2. Preparation and Training

In the week prior to the study, all ED triage nurses received standardized information on frailty and were trained to score the three screening instruments. While many of the ED nurses had some knowledge of frailty, this was not uniform. Education sessions were conducted between hand-over sessions to include those coming on the day shift and off the night shift. Training was study-specific and mainly aimed at explaining the nature of the study and what would be expected of ED staff to help facilitate the project. Inter-rater reliability (IRR) was completed for a small sample (*n* = 20) of patients.

### 2.3. Screening Instruments

The Rockwood Clinical Frailty Scale (CFS) is a brief, global, subjective frailty scale, often used to stratify patients after a clinical assessment [30,31,32]. It is widely used and has been validated in ED to predict increased length of stay (LOS), inpatient mortality, and admission to a geriatric unit but not readmission [2]. It has also been used to predict LOS in hospital medicine units [31]. The CFS combines pictographs and written descriptors (nine-point version) and is scored from one (very fit) to nine (terminally ill). Those scoring four are considered vulnerable (pre-frail) and those scoring five (mild) to eight (very severely) are considered frail. It can be corrected for people with dementia [30]. The PRISMA-7 consists of seven dichotomous yes/no answers covering age, gender, general health (two questions), activities, and social supports. One point is scored for each of the seven questions and a cut-off score of ≥3 points suggests the need for further assessment. It can be used as a postal or telephone questionnaire [25]. The Identification of Seniors at Risk (ISAR) is a self-reported six-item questionnaire again using a yes/no format that measures care needs before and after an acute illness, number of hospital admissions, vision, memory, and medication use [33]. Validated for use in the ED, it is scored from zero to six with a cut-off score of ≥2 considered as high-risk [33]. While primarily developed as a risk-prediction tool, which may not necessarily equate with a frailty screen [23,33], it is accurate with high sensitivity in identifying frailty in the ED [11], though less accurate in predicting specific adverse healthcare outcomes in this setting [34,35,36].

### 2.4. Data Collection and Study Outcome

Screening was conducted after the standard ED triage was completed. The triage nurse on duty scored patients with the CFS [30]. Patients were then asked to complete two self-reported instruments: the ISAR tool [33] and the PRISMA-7, administered by the nurses or during busy periods by a research assistant, in alternative order to minimize fatigue effects. Where patients were deemed unable to comply due to sensory or cognitive impairment, caregivers, family, or those attending with patients, where available and with permission of the participant, were invited to assist. Test scores of all three instruments were then concealed. A dedicated multi-disciplinary frailty team including a geriatric consultant completed a CGA (described below) on all those screened, blind to the screening test scores. The consultant adjudicated all cases to ensure quality control. Medical records and medication lists were reviewed and a battery of standardised assessments was completed within 24 h if admitted. Where available, a collateral history was obtained and family members asked to complete a Caregiver Burden Score. Those not requiring admission were prioritised for CGA prior to discharge home. Outcomes were frailty based on the CGA and as measured with the FRAIL scale and GFI. A provision to follow-up patients screened at triage but discharged from ED before CGA could be completed by telephone was requested in the ethics application.

### 2.5. Comprehensive Geriatric Assessment to Determine Frailty Status

GCA was used in this study as a “gold standard” for measuring and confirming whether patients were frail or not, independent of the screening tests under examination i.e., frailty status (frail or non-frail) was based on the clinical judgment of a consultant geriatrician following a CGA supported by two validated frailty measures: the FRAIL Scale [37] and the Groningen Frailty Indicator (GFI) [38]. The FRAIL scale, ranging from 0 (not frail) to 5 (most frail), was used to help classify pre-frailty taking a score of 1 or 2 as pre-frail and >2 as frail. The GFI is a 15-point yes/no questionnaire exploring physical, cognitive, social, and psychological features of frailty. It was used to evaluate frailty domains with a cut-off of ≥4/15 identifying moderate–severe frailty. Nutrition was further assessed with body mass index (BMI) and the Mini-Nutritional Assessment-short form (MNA-SF) [39]. The MNA-SF is validated in frailty and a cut-off score of ≤11 identified those at risk of malnutrition [40]. Cognition was screened with the Alzheimer’s Disease 8 (AD8) [41], completed with collateral. If no caregiver was present, the patient was asked to complete the AD8 (pAD8). Validated for detecting cognitive dysfunction among older adults in the ED [42], a score of ≥2 suggests cognitive impairment. The abbreviated mental test score (AMTS), taking a cut-off of seven (<7/10) [43], was used as a brief test to support the diagnosis for those screening positive. Quality of life was measured with the Euroqol EQ-5D [44], including its visual analogue scale (VAS) from 0 (worst imaginable health state today) to 100 (best imaginable). Self-rated health status was inferred from a single item on the SF-36 instrument (“*In general, would you say your health is, excellent; very good; good; fair or poor?*”) [45]. Carer strain was evaluated using Caregiver Burden Score (CBS) [46], a shorter version of the Zarit Burden Interview. The CBS is composed of six questions and describes the degree to which caring affects the caregiver from 0 (none of the time) to 6 (all of the time). Completed by the caregiver, CBS scores of ≥15/30 suggest burden and ≥25/30 suggest severe burden or burnout.

### 2.6. Statistical Analysis

Data were analysed with SPSS V21.0 (IBM, Chicago, IL, USA). The Shapiro–Wilk test was used to test normality and found that most data were non-parametric. Spearman’s correlation coefficient was used to assess IRR. The Mann–Whitney U test compared non-parametric samples. Binary logistic regression was used to explore the strength of the relationship between variables. The sensitivity, specificity, positive predictive value (PPV), and negative predictive value (NPV) of each screen were calculated at different cut-offs. Accuracy was assessed from the area under the curve (AUC) of receiver operating characteristics (ROC) curves, compared with the DeLong method [47]. Agreement between the index tests and reference standards were calculated using Cohen’s kappa. Based upon data that suggests the PRISMA-7 has a sensitivity of 0.83 and specificity of 0.83 [26], a power calculation estimating an expected prevalence of frailty among those ≥70 attending ED of 50% (limited data were available for this population, though a recent paper suggests that 60% aged ≥65 are frail) [11], at a significance level of 0.05, a power of 80% and an effect size of 10%, yielded a recommended sample size of 275 participants [48]. The optimal cut-off was calculated using Youden’s Index (J = Sensitivity + Specificity − 1).

## 3. Results

In all, 307 patients were available over two weeks of screening. This represents 76% (307/403) of all those aged ≥70 attending ED during this period. Of these, 265 were included in this analysis: 15 were screened but had incomplete data and 27 were excluded because they were lost to follow-up (*n* = 10), decompensated prior to or during the assessment (*n* = 7), declined to participate (*n* = 5), unable to participate (*n* = 3), or non-English speaking (*n* = 2). The characteristics of those included are presented in Table 1.

The median age (interquartile range ±) of patients included was 78 (83−74 = ±9) years of which most were female (54%). Those excluded were younger (median 76 years ±9, *z* = −2.4, *p* = 0.02) but had a similar gender profile (52% female, *p* = 0.88). Based upon the CGA, 58% (154/265) of patients were classified as frail, the remainder as non-frail (robust or pre-frail). Frail patients were statistically significantly older (*p* = 0.001) and had lower median MNA-SF (*p* < 0.001), AMTS (*p* = 0.04), GSRH (*p* < 0.001), and EQ-5D VAS (*p* < 0.001) scores than non-frail patients. They also had higher median AD8 scores (*p* < 0.001) and CBS scores reported by a collateral (*p* < 0.001). Using the FRAIL scale, 31% of patients were classified as robust, 41% pre-frail, and 28% frail. Based on the GFI, 58% were moderate to severely frail. Correlation between FRAIL Scale and the GFI was moderate and positive *r* = 0.5 with 64% agreement.

**Figure 1:**
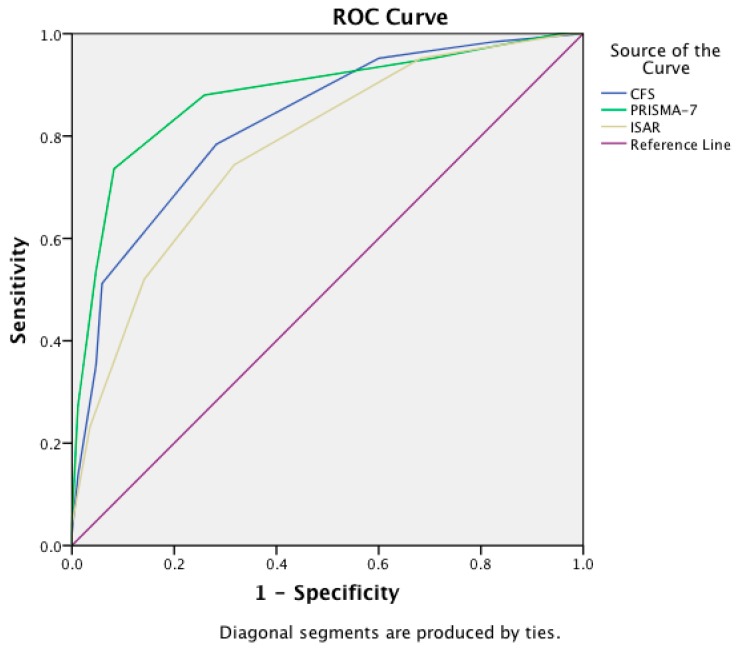
Receiver operating characteristic curves with area under the curve scores showing the accuracy of the Clinical Frailty Scale (CFS), Programme of Research to Integrate Services for the Maintenance of Autonomy 7 (PRISMA-7) tool, and the Identification of Seniors at Risk (ISAR) tool in differentiating frail from non-frail older adults in the Emergency Department (classification based on the **comprehensive geriatric assessment**).

**Figure 2:**
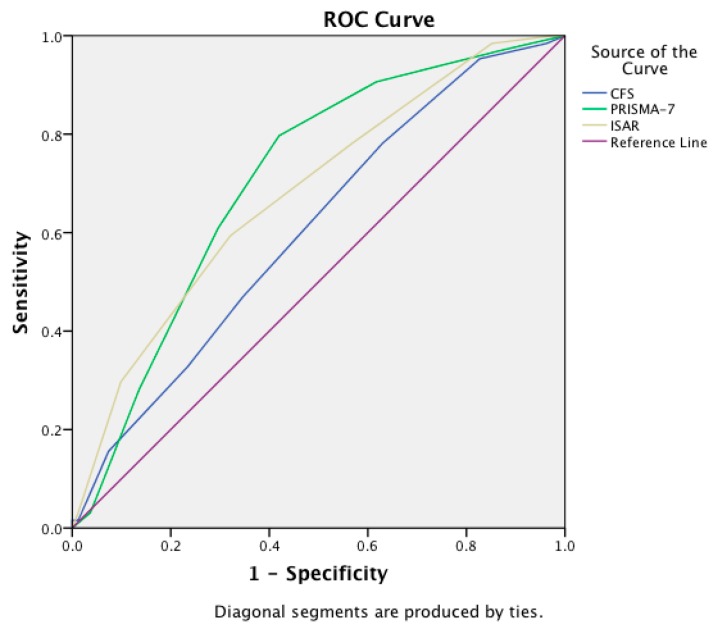
Receiver operating characteristic curves with area under the curve scores showing the accuracy of the Clinical Frailty Scale (CFS), Programme of Research to Integrate Services for the Maintenance of Autonomy 7 (PRISMA-7) tool, and the Identification of Seniors at Risk (ISAR) tool in differentiating frailty from pre-frailty (classification based on the **FRAIL scale**).

The IRR of the instruments varied from moderate to strong: ISAR (*r* = 0.62), CFS (*r* = 0.78), and PRISMA-7 (*r* = 0.75). The questionnaires (PRISMA-7 and ISAR) were mainly scored by the patient themselves (82%), the remainder were administered to patient’s caregivers or by the nurses most often because of cognitive or visual impairment. The median ISAR score was 3 (4−2 = ±2), median CFS was 4 (5−3 = ±2), and PRISMA-7 was 3 (5−2 = ±3). The most accurate instrument for separating frail from non-frail patients (based on the CGA determination) was the PRISMA-7 (AUC of 0.88; 95% Confidence Interval (CI): 0.83–0.93), followed by the CFS (AUC of 0.83; 95% CI: 0.77–0.88), and the ISAR (AUC of 0.78; 95% CI: 0.71–0.84), see Figure 1. The PRISMA-7 was statistically significantly more accurate than the ISAR (*p* = 0.008) but not the CFS (*z* = 1.4, *p* = 0.15). The PRISMA-7 was also the most accurate at differentiating pre-frail from frail using the FRAIL scale to measure pre-frailty, (AUC of 0.71; 95% CI: 0.62–0.79), see Figure 2.

The diagnostic accuracy (AUC of ROC curves) comparing all three approaches to measuring frailty are presented in Table 2. This shows that irrespective of the frailty classification approach used, the PRISMA-7 had the highest accuracy.

At its recommended cut-off score (≥2) the ISAR had excellent sensitivity (95%) but poor specificity (35%) for detecting frailty based on the CGA; a cut-off of ≥3 provided greater sensitivity (72%) while retaining reasonable specificity (72%). The CFS taking a score of ≥5 for frailty based on the CGA had poor sensitivity (51%) but excellent specificity (94%). The PRISMA-7 had the best balance at its recommended cut-off (≥3) with good-excellent sensitivity (84%) and specificity (78%) for frailty as determined by CGA. Sensitivity, specificity, NPV, PPV, and false positive and negative rates for detecting frailty based on the CGA for each of the three screens at a range of cut points are presented for reference in Table 3. Note that low scores on all three screens reduced the sensitivity for frailty while higher scores increased the specificity for frailty. The optimal cut-offs based on the data here were ≥4, ≥4, and ≥3 for the CFS, PRISMA-7, and ISAR, respectively. The FRAIL scale was more sensitive (100%), albeit with lower specificity (58%) at a cut-off of ≥3 for frailty based on the CGA than the GFI, (sensitivity 93%, specificity 88%) at a cut-off of ≥4 for moderate to severe frailty.

## 4. Discussion

This study presents a clinimetric evaluation and comparison of three brief frailty screening and risk-prediction instruments, the CFS, ISAR, and PRISMA-7 in an ED setting. To our knowledge, it is the first validation of the widely-used and endorsed PRISMA-7 in the ED, where despite its inclusion in frailty pathways and several policy statements, it had yet to be validated [18]. The results suggest that the PRISMA-7 is the most accurate in identifying frailty as confirmed by CGA, albeit was not statistically significantly better than the CFS. It had the best balance between sensitivity and specificity at its usual cut-off (≥3), which were similar to that reported in the community [27]. It also had strong concordance among raters, albeit all three instruments had moderate to strong IRR. The results reflect studies showing that the ISAR has only fair accuracy in the ED [36]. At its usual cut-off score (≥2) it had excellent sensitivity but particularly poor specificity and a high false-positive rate (33%). A higher cut-off (≥3) offered the best balance between sensitivity and specificity. The accuracy of the CFS suggests that it could be used by non-specialists (triage nurses) as a visual analogue scale to quickly classify patients rather than solely as a post CGA stratification instrument, particularly in a busy setting such as ED where additional lengthy screening instruments may not be feasible or desirable to all [18]. However, its sensitivity for detecting frailty was low, taking a cut-off of five for established frailty and its specificity was high, suggesting that if time allows, it should ideally be used in conjunction with another frailty screen with higher sensitivity. As all three instruments have administration times less than five minutes [19], this could be a feasible approach.

A strength of this study is the sampling approach, which unlike similar studies [19] attempted to recruit consecutive patients. The CGA, while targeted, was broad and completed by a specialised frailty MDT led by a consultant geriatrician, taking a multi-domain approach to diagnosing frailty. There were several limitations to this study. As consecutive screening was important to achieve a representative sample and reach the requisite sample size, every attempt was made to ensure screening continued outside of daytime shift hours. While largely successful, during busy triage periods or during acute emergencies, especially during the on-call night hours when staffing levels were lower, some patients who may otherwise have been suitable were not screened. No data on those not screened were available. Further, some patients attending ED during the night but not requiring admission, despite being screened at triage were lost to follow-up. These challenges are reflected in the missing screening and assessment data, where some but not all scores were completed for each individual. This was anticipated in advance, accounted for in the sample size calculation, and an attempt was made to follow-up all patients who had incomplete data by telephone. In addition, the use of the MTS may have led to over-triaging, characterising patients are more acutely unwell than [49], excluding patients who would otherwise have met inclusion criteria. Nevertheless, the MTS is widely used and is the recommended in-hospital prioritisation system in Irish EDs [50]. The use of different types of instruments, the CFS which is a subjective assessment by a trained rater based on available information and two questionnaires (ISAR and PRISMA-7) may have affected the comparison. Both approaches to screening have limitations. The use of self-reported instruments may have reduced the accuracy of results (reporting bias), particularly when scored with patients with cognitive impairment, though in this study the two instruments that could be self-administered were in many cases scored by staff with patients and or their families; these were also more accurate than the subjective CFS scored by the nurses, suggesting that the impact of reporting bias was minimal. Nevertheless, future studies should consider other approaches to identifying frailty in ED such as the Modified Bournemouth Criteria [51]. Similarly, while the reliability of the screens was good, the IRR was not strong for all three instruments. Another limitation is that the CGA itself is not a gold standard and included scales are not in themselves recognised as gold standards e.g., the AMTS is insensitive to early cognitive changes and likely under-appreciated the prevalence of cognitive impairment. This may have affected (reduced) the validity of the CGA. However, scales were selected for practical considerations and were only used to support the diagnosis made by the expert geriatrician where available. Further, the goal was to identify frailty states rather than prodromal conditions such as mild cognitive impairment or screen for occult conditions such as dementia. Finally, while the ED studied is broadly representative of EDs in Ireland, it may differ in the number and type of older adults presenting, potentially reducing the generalisability of the findings, particularly to other health care systems in other countries.

While a clear rationale for identification of frailty through opportunistic screening at the beginning of each urgent care episode is emerging [15], not all older patients receive this in the ED [19]. This means that suitable patients may not benefit from early risk stratification, prompt CGA, entry to dedicated frailty pathways, and tailored interventions where these are available. This study showed that the PRISMA-7 at its recommended cut-off score has good to excellent accuracy and that it had the best balance between sensitivity and specificity of these three commonly used instruments, supporting its inclusion in recent recommendations including the BGS’s Fit For Frailty guidance document [24]. Further study is now needed to confirm this and compare the PRISMA-7 with other short frailty screening instruments including those designed specifically for use in busy EDs exploring acceptability and feasibility as well as diagnostic test accuracy. Investigating new or combinations of existing brief instruments for use in the ED to maximise sensitivity and specificity may also be useful.

## 5. Conclusions

In summary, this paper compares three commonly-used frailty screening instruments to identify frailty at the interface between community and hospital care, the ED. The results show that all of these short scales are reliable and accurate to score in ED with the PRISMA-7 being more accurate than the ISAR and having equivalent accuracy to the CFS. Given the relatively subjective nature of the CFS, particularly when used in this setting (ED triage), where staff only have a brief impression of patients, screening with the PRISMA-7 may be optimal. The PRISMA-7 is a questionnaire and while it depends on the accuracy of respondents, it requires less training and could be easier to administer. Nevertheless, both could be used at ED triage to identify older adults who are frail and in need of targeted interventions to mitigate the risk of adverse outcomes. As it is known that frailty status at admission to hospital predicts adverse outcomes, it is important to identify frailty as early into an admission as possible [15]. Such triage could improve the efficiency of acute hospital services for older people by allowing the cohorting of appropriate patients to older person’s or frailty-specific units, by promoting early allocation of CGA that can reduce mortality and institutionalisation and by facilitating re-direction to more appropriate care in the community such as hospital at home or day hospital services [52]. Given the growing importance of frailty from a public health perspective [53], the as-yet limited interventions to avoid delayed discharges or hospital readmissions [54] and the challenges of delivering integrated care to older people across settings [55], identifying frailty so early could make acute and community care [56] more responsive for all patients.

## Figures and Tables

**Table:** 

Instrument	Area Under the Curve	95% Confidence Interval
CFS	0.83	0.77–0.88
PRISMA-7	0.88	0.83–0.93
ISAR	0.78	0.71–0.84

**Table:** 

Instrument	Area Under the Curve	95% Confidence Interval
CFS	0.61	0.51–0.70
PRISMA-7	0.71	0.62–0.79
ISAR	0.68	0.59–0.77

**Table 1:** Characteristics of patients (*n* = 265) screened for frailty comparing those classified as frail and non-frail (based on the comprehensive geriatric assessment classification).

Predictor	Total Median (Q3−Q1 = ±IQR)	Frail Median (Q3−Q1 = ±IQR)	Non Frail Median (Q3−Q1 = ±IQR)	P = X
Age (Years)	78 (83−74 = ±9)	80 (84−75 = ±9)	76 (82−73 = ±9)	*z* = −3.3 *p* = 0.001
Sex (% Female)	54%	54%	53%	X^2^(1) = 0.01 *p* = 0.91
BMI	25.7 (28.7−22.5 = ±6.2)	25.3 (28−22 = ±6)	25.9 (29−23 = ±6)	*z* = −1 *p* = 0.36
MNA-SF	11 (13−9 = ±4)	9 (12−7 = ±5)	12 (14−11 = ±3)	*z* = −7.5 *p* < 0.001
AD8	0 (2−0 = ±2)	1 (3−0 = ±3)	0 (0−0 = ±0)	*z* = −6.8 *p* < 0.001
AMTS	9 (10−7 = ±3)	9 (10−5 = ±5)	10 (10−8 = ±2)	*z* = −2 *p* = 0.04
CBS	4 (17−0 = ±17)	12 (20−4 = ±16)	0 (0−0 = ±0)	*z* = −4.5 *p* < 0.001
EQ-5D (VAS)	60 (80−50 = ±30)	50 (60−40 = ±20)	80 (85−60 = ±25)	*z* = −8.3 *p* < 0.001
GSRH (% Very good or excellent)	21%	5%	43%	X^2^(1) = 53 *p* < 0.001
GFI	4 (7−2 = ±5)	6 (8−5 = ±3)	2 (3−1 = ±2)	*z* = −12.4 *p* < 0.001
FRAIL scale	1 (3−0 = ±3)	2 (3−1 = ±2)	0 (1−0 = ±1)	*z* = −10.1 *p* < 0.001
ISAR	3 (4−2 = ±2)	3.5 (4−2 = ±2)	2 (3−1 = ±2)	*z* = −8.1 *p* < 0.001
CFS	4 (5−3 = ±2)	5 (6−4 = ±2)	3 (4−2 = ±2)	*z* = −8.2 *p* < 0.001
PRISMA-7	3 (5−2 = ±3)	5 (6−3 = ±3)	2 (2−1 = ±1)	*z* = −10.3 *p* < 0.001

BMI—Body Mass Index; MNA-SF—Mini-Nutritional Assessment-short form; AD8—Alzheimer’s Disease 8; AMTS—Abbreviated mental Test Score; CBS—Caregiver Burden Score; EQ-5D-VAS—Euroqol EQ-5D Visual Analogue Scale; General Self-Rated Health—GSRH; GFI—Groningen Frailty Indicator; ISAR—Identification of Seniors at Risk; CFS—Clinical Frailty Scale; PRISMA-7—Programme of Research to Integrate Services for the Maintenance of Autonomy 7.

**Table 2:** Diagnostic accuracy based on the area under the curve (AUC) values with 95% confidence intervals (CI) for the Clinical Frailty Scale (CFS), Programme of Research to Integrate Services for the Maintenance of Autonomy 7 (PRISMA-7) tool, and the Identification of Seniors at Risk (ISAR) tool for separating frail and non-frail based on (1) the comprehensive geriatric assessment (CGA), (2) FRAIL scale, and (3) Groningen Frailty Indicator (GFI) frailty classification approaches.

Frailty Classification Approach	CGA AUC (with 95% CI)	FRAIL Scale AUC (with 95% CI)	GFI AUC (with 95% CI)
**Instrument**			
CFS	0.83 (0.77–0.88)	0.68 (0.61–0.76)	0.81 (0.75–0.86)
PRISMA-7	0.88 (0.83–0.93)	0.79 (0.74–0.85)	0.82 (0.77–0.87)
ISAR	0.78 (0.71–0.84)	0.74 (0.67–0.80)	0.77 (0.71–0.82)

**Table 3:** Sensitivity, specificity, positive predictive value (PPV), and negative predictive value (NPV), with 95% confidence intervals (CI), for the Clinical Frailty Scale (CFS), Programme of Research to Integrate Services for the Maintenance of Autonomy 7 (PRISMA-7) tool, and the Identification of Seniors at Risk (ISAR) tool for frail and non-frail (based on the **comprehensive geriatric assessment** classification).

Frailty Screen Cut-Off Score	Youden’s Index	Sensitivity (95% CI)	Specificity (95% CI)	PPV (95% CI)	NPV (95% CI)	False Positive (95% CI)	False Negative (95% CI)
**CFS**							
≥3	0.35	95% (89–98)	40% (30–51)	70% (62–77)	85% (69–94)	30% (23–38)	15% (6–31)
≥4 ^	0.5	78% (70–85)	72% (61–81)	80% (72–87)	69% (58–78)	20% (13–28)	31% (22–42)
≥5 *	0.45	51% (42–60)	94% (86–98)	93% (83–97)	57% (48–65)	7% (3–17)	43% (35–52)
≥6	0.3	35% (27–44)	95% (88–98)	92% (79–97)	50% (42–58)	8% (3–21)	50% (42–58)
**ISAR**							
≥1	0.05	100% (97–100)	5% (2–11)	59% (53–65)	100% (46–100)	41% (35–47)	0% (0–54)
≥2 *	0.3	95% (90–98)	35% (26–45)	67% (60–73)	83% (69–92)	33% (27–40)	17% (8–31)
≥3 ^	0.44	72% (64–79)	72% (63–80)	78% (70–84)	65% (56–73)	22% (16–30)	35% (27–44)
≥4	0.38	50% (42–58)	88% (80–93)	86% (76–92)	56% (48–63)	14% (8–24)	44% (37–52)
≥5	0.17	20% (14–28)	97% (92–99)	91% (75–98)	53% (47–60)	9% (2–25)	53% (47–60)
**PRISMA-7**							
≥2	0.24	94% (88–97)	30% (22–39)	65% (58–71)	77% (61–88)	35% (29–42)	23% (12–39)
≥3 *	0.62	84% (77–90)	78% (69–85)	84% (77–90)	78% (69–85)	16% (10–23)	22% (15–31)
≥4 ^	0.63	70% (62–77)	93% (86–97)	93% (86–97)	69% (61–76)	7% (3–14)	31% (24–39)
≥5	0.47	51% (43–59)	96% (90–99)	95% (87–98)	58% (51–66)	5% (2–13)	42% (34–49)
≥6	0.24	25% (19–33)	99% (94–100)	98% (85–100)	49% (42–56)	3% (0–15)	51% (44–58)

* recommended cut-off score for frailty, ^ optimal cut-off based on Youden’s Index.
